# Polarized SCAR and the Arp2/3 complex regulate apical cortical remodeling in asymmetrically dividing neuroblasts

**DOI:** 10.1016/j.isci.2023.107129

**Published:** 2023-06-15

**Authors:** Giulia Cazzagon, Chantal Roubinet, Buzz Baum

**Affiliations:** 1 Medical Research Council Laboratory of Molecular Biology, Cambridge CB2 0QH, UK

**Keywords:** Biological sciences, Molecular biology, Neuroscience, Cell biology

## Abstract

Although the formin-nucleated actomyosin cortex has been shown to drive the changes in cell shape that accompany animal cell division in both symmetric and asymmetric cell divisions, the mitotic role of cortical Arp2/3-nucleated actin networks remain unclear. Here using asymmetrically dividing *Drosophila* neural stem cells as a model system, we identify a pool of membrane protrusions that form at the apical cortex of neuroblasts as they enter mitosis. Strikingly, these apically localized protrusions are enriched in SCAR, and depend on SCAR and Arp2/3 complexes for their formation. Because compromising SCAR or the Arp2/3 complex delays the apical clearance of Myosin II at the onset of anaphase and induces cortical instability at cytokinesis, these data point to a role for an apical branched actin filament network in fine-tuning the actomyosin cortex to enable the precise control of cell shape changes during an asymmetric cell division.

## Highlights

• Polarized SCAR and Arp2/3 activity set the stage for asymmetric fly stem cell divisions
• Arp2/3 controls the dynamics of cortical remodeling in cells entering anaphase
• Apical SCAR recruitment and protrusion formation depend on the polarity protein Pins
• Arp2/3 and SCAR limit the apical accumulation of Myosin as cells enter anaphase

## Graphical abstract

**Figure:**
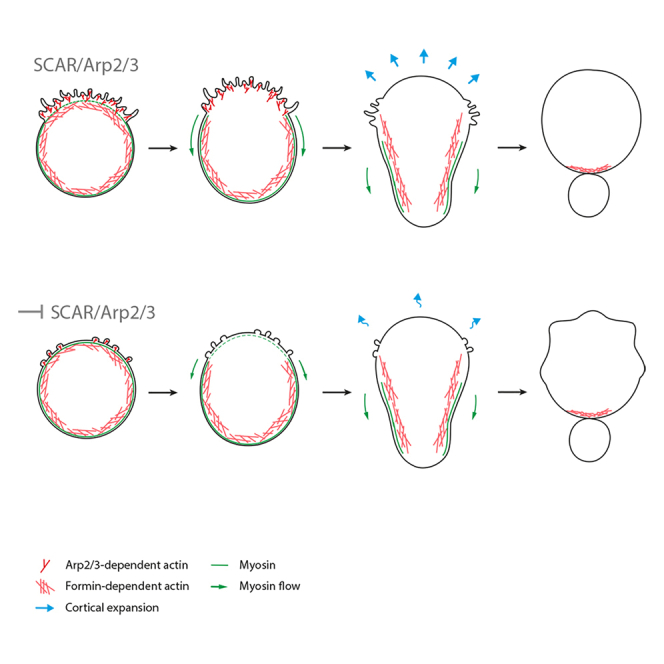

## Introduction

A predominantly formin-nucleated actomyosin cortex is known to control the changes in cell shape that accompany division. However, much remains to be discovered about the role of branched actin networks in this process. The Arp2/3 complex is a nucleator of actin branched filaments, best known for its role in the formation of lamellipodial protrusions during adherent cell spreading and migration, in intracellular motility of pathogens, and in the fission of membranes during trafficking.^1^^,^^2^^,^^3^

It has been previously suggested that the Arp2/3 complex is mostly active in interphase, playing limited roles during mitotic entry and mitotic exit.^4^ As examples of this, Arp2/3-dependent actin filaments have been shown to form at centrosomes in mitotic cells and at the interface between newly divided cells.^5^^,^^6^^,^^7^^,^^8^^,^^9^ In addition, a growing body of work carried out using mammalian cells in culture has suggested that the Arp2/3 complex can also generate actin filaments during mitosis. Indeed, in HeLa cells, the Arp2/3 complex has been shown to induce the formation of a rotating wave of actin filaments^10^^,^^11^. Furthermore, it has recently been shown that Arp2/3 dependent actin filament formation modulates cortical mechanics to aid asymmetric cell divisions in both *C. elegans* and *Drosophila*.^12^^,^^13^ However, it remains unclear whether or not the Arp2/3 complex plays additional functions during mitosis.

In general, the mechanisms that lead to shape changes in dividing *Drosophila* cells are very similar to those operating in vertebrate cells. In brief, upon entry into mitosis, the activation of Pbl/Ect2 triggers formin-dependent actin filament formation along with non-muscle Myosin II activation (hereafter called Myosin) to generate a contractile mitotic actomyosin cortex, which drives mitotic rounding.^4^^,^^14^^,^^15^^,^^16^ Then, at mitotic exit, cues from the spindle midzone and, more controversially, the anaphase chromatin, polarize the mitotic cell cortex to allow for the formation of a contractile actomyosin ring and division.^4^^,^^14^^,^^17^^,^^18^ Thus far, however, only a few studies have addressed the role of the Arp2/3 complex in mitotic cortical remodeling.^9^^,^^12^^,^^13^

Cells undergoing asymmetric divisions, like *Drosophila* neuroblasts, are likely to face additional challenges as they divide. At each round of asymmetric division, these neuronal stem cells produce two daughter cells that differ in both size and fate: a larger cell which retains stem cell identity, and a smaller cell, known as a ganglion mother cell (GMC), which divides again to give rise to two differentiated neural or glial cells.^19^^,^^20^ The establishment of the physical asymmetry that underlies asymmetric division in this system therefore also depends on the action of cortical polarity cues. As cells enter anaphase, these lead to asymmetric remodeling of the actomyosin cortex, causing Myosin II to flow away from the apical cortex before it is cleared from the basal cortex. This leads to biased cortical expansion, and to a basal shift in the positioning of the division ring, which in turn leads to an asymmetric division that generates unequally sized sibling cells.^21^^,^^22^^,^^23^

These actomyosin flows have been associated with the movement of PIP_2_-rich membrane domains.^24^^,^^25^^,^^26^ Thus, in neuroblasts, apically directed flows of PIP_2_ lipids were observed using PH-domain probes, which were reversed at the onset of anaphase, leading to the dispersal of the membrane domains across the cell surface as cells underwent apical expansion.^24^ The movements of these PIP_2_-rich domains were also shown to depend on cortical polarity and on the actomyosin cortex, implying a link between the membrane and the underlying cortex that could be important for asymmetric division.^24^^,^^25^^,^^26^^,^^27^

Here, in this paper, we explore the roles of the Arp2/3 complex and its upstream nucleation promoting factors in the regulation of membrane remodeling, mitotic cell shape, and division in fly neuroblasts. Using a combination of genetics, RNAi, small molecule inhibitors and live cell imaging, our analysis reveals the existence of a pool of polarized actin-based membrane protrusions at the apical side of mitotic neuroblasts, which co-localize with SCAR complex components, whose organization depends on the activity of SCAR and the Arp2/3 complex. This local remodeling of the actin cortex appears to limit apical Myosin accumulation in metaphase and, when perturbed, leads to cortical defects and membrane instability at cytokinesis. Taken together, these data suggest that the accumulation of an apical Arp2/3-dependent branched actin network may polarize the actomyosin cortex in mitotic neuroblasts to help guide the precisely choreographed cortical remodeling necessary for asymmetric cell division.

## Results

**Figure 1:**
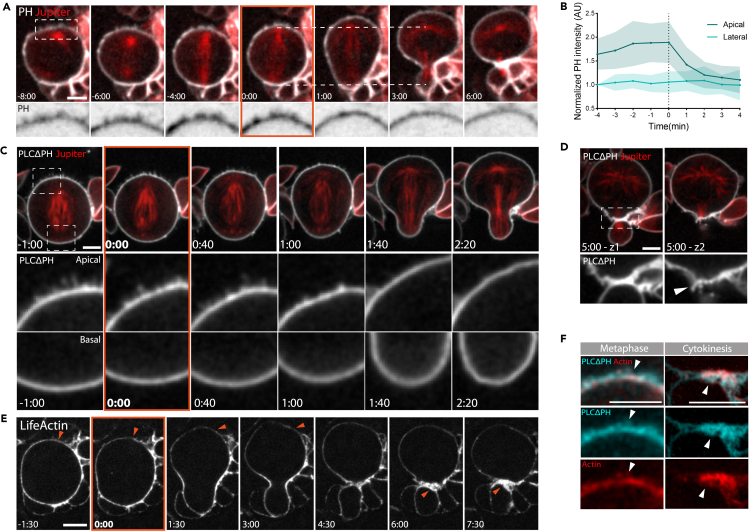
Mitotic neuroblasts exhibit polarized membrane protrusions that disappear with cortical expansion following the onset of anaphase (A) Representative images of dividing neuroblasts expressing a PLCΔPH::GFP membrane marker and an mCherry:Jupiter microtubule marker via Wor-GAL4/UAS. Dotted lines highlight cortical expansion of the apical (but not basal) domain. The region in the dotted box is highlighted in the Zoom below. (B) Plot shows PLCΔPH::GFP intensity at the apical or lateral membrane of neuroblasts progressing through mitosis. Data were normalized by subtracting the background and by dividing all data points by PH intensity at the lateral domain at time 4 min before the onset of anaphase (−4 min). Central and error bars: mean and standard deviation. n = 10. (C) Super-resolution imaging of a representative neuroblast expressing PLCΔPH::GFP and mCherry:Jupiter. Inserts show apical and basal domains, respectively. (D) Image shows consecutive Z-slices through a dividing neuroblast. Arrowhead points to membrane protrusions at the furrow. (E) A representative neuroblast expressing actin reporter UAS-LifeAct:GFP as it divides. Arrowheads point to actin filaments being cleared from the apical cortex during metaphase-anaphase transition (−1:30 to 3:00 min), and to the later accumulation of actin at the cytokinetic furrow (6:00-7:00 min). (F) Super-resolution stills of live neuroblasts at different stages of mitosis in which Wor-GAL4/UAS has been used to express PLCΔPH:mCherry to label the membrane and LifeAct:GFP to mark actin filaments. Arrowheads point to actin-rich membrane protrusions. Scale bars = 5 μm.

To study changes in membrane organization during passage through mitosis we began by using the Pleckstrin Homology (PH) domain from the phospholipase CΔ1 (PLCΔ1) that interacts with the head group of the phosphatidylinositol 4,5-bisphosphate (PIP_2_) as a probe. This marker was chosen because it labels the plasma membrane and membrane protrusions, but not internal membranes (Figure 1A). Through this analysis, we observed changes in apical membrane organization that accompany mitotic progression (Figure 1A). When we compared the intensity of the PLCΔ1:GFP signal at the apical domain and at the lateral membrane of mitotic cells, we saw that the apical GFP signal increases from prophase to metaphase and then rapidly decreases upon the onset of anaphase, while the signal at the lateral membrane remains constant (Figures 1B, and S1A and S1B). Furthermore, as previously reported, an analysis of bright PH-labelled membrane domains also revealed a change in the direction of membrane domains flows that depends on the cell cycle stage (Figure S1C).^24^^,^^25^

To better understand how these flows of PLCΔ1:GFP are changed in fly neuroblasts at the metaphase-anaphase transition, we used super-resolution spinning-disk confocal microscopy to image cells at a higher temporal (20 s/frame) and spatial resolution. In these movies, when the apical surface of cells was not in contact with overlying tissue, filopodia-like membrane structures could be seen forming at the apical surface of metaphase cells (Figure 1C, Apical insert, −1:00 to 0:40 min), whereas the basal cortex remained relatively unchanged and smooth over the same period (Figure 1C, Basal inserts). These apical protrusions were 0.7–1 μm in length, started to disappear 1 min after anaphase onset, concurrently with apical expansion, and were completely gone by the end of telophase (Figure 1C, Apical insert, 2:20 min). These protrusions were also visible at the apical surface of cells using a second membrane marker, GAP43 (Figure S1D), demonstrating that they are a characteristic feature of the apical membrane independently of the reporter used. Importantly, at the end of cell division, an additional population of protrusions visible using the same markers was seen forming between the two daughter cells at the site of cleavage (Figure 1A and 1D, arrowhead), similar to those described previously at new cell interfaces in other cell types.^6^^,^^8^^,^^28^

Because most membrane protrusions in animal cells are actin-based, we looked for the presence of actin filaments in these structures using LifeAct:GFP.^29^ This revealed an accumulation of actin at the apical cortex, which was cleared during the metaphase-anaphase transition (Figure 1E, arrowheads from −1:30 to 3:00 min, Figure 1F, Metaphase arrowheads). Later, a second population of actin-rich protrusions appeared at the furrow following cytokinesis (Figure 1E, arrowheads 6:00-7:30 min). Both sets of actin structures partially colocalized with PH::GFP in flies carrying both markers (Figure 1F, arrowheads).

**Figure 2:**
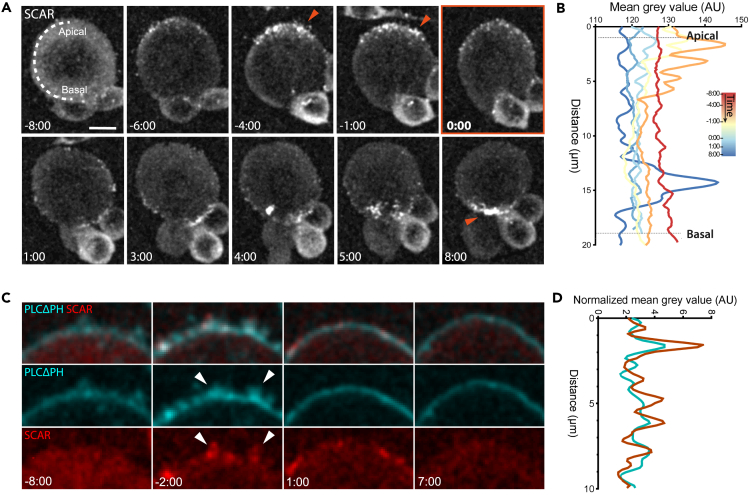
SCAR co-localizes with membrane protrusions in dividing neuroblasts (A) Representative super-resolution maximum z-projection of dissociated neuroblast expressing SCAR::GFP driven by Wor-GAL4/UAS. Arrowheads point to the accumulation of SCAR at the apical side of the neuroblast in metaphase, and at the furrow in cytokinesis. n = 15 for cells in the whole brain, n = 3 for isolated cells (as in image). (B) Graph showing mean SCAR::GFP intensity acquired by drawing a line around the cortex from the apical to the basal side - as depicted at time −8:00 in the image in A. Individual intensity profiles are color-coded by time as indicated. (C and D) Super-resolution imaging of a representative neuroblast showing the partial co-localization of membrane protrusions (UAS-PH:mCherry) and SCAR (UAS-SCAR::GFP). n = 5 D. Graph showing average PH and SCAR signals over time, obtained by drawing a line along the portion of cortex as shown in the insert at −2:00 min before anaphase onset. Data were normalized by subtracting background. Scale bars = 5 μm.

Because actin-based membrane protrusions at the cytokinetic furrow of *Drosophila* sensory organ precursors (SOPs) have previously been shown to depend on the actin nuclear-promoting factor SCAR,^9^^,^^30^ we decided to test the involvement of SCAR in the generation of apical protrusions in neuroblasts. To do so, we first dissociated larval brains to look at isolated neuroblasts, and imaged cells expressing SCAR::GFP at high spatial resolution. In this experiment, SCAR was specifically seen accumulating to high levels at the apical side of the metaphase cortex (Figure 2A, arrowheads, and Figure 2B). When the neuroblast entered anaphase, SCAR::GFP was then lost from the apical surface, before accumulating at the basal side of the cell sometime later, where it became concentrated at the cytokinetic furrow (Figure 2A, arrowhead, and Figure 2B). Because SCAR is part of a stable multiprotein complex,^1^ we were able to confirm its localization using a second complex component, Abi (Figure S2). To follow the dynamic pattern of SCAR localization through mitosis in more detail, we next imaged PH:mCherry and SCAR::GFP in parallel in cells expressing both probes (Figure 2C). Although the fluorescent signal appeared low in neuroblasts in prophase (Figure 2C, −8:00 min), by metaphasenumerous apical SCAR-positive membrane protrusions were observed (Figure 2C, −2:00 min, arrowheads), whose localization largely overlapped with PH-rich structures in an intensity profile drawn along the apical cell surface. At anaphase, most of these SCAR-rich punctae then disappeared as the apical cortex expanded, along with most surface protrusions, as measured by the PH:mCherry signal. However, a few SCAR::GFP punctae remained visible on the apical surface of cells 1 min after anaphase onset (Figure 2C).

**Figure 3:**
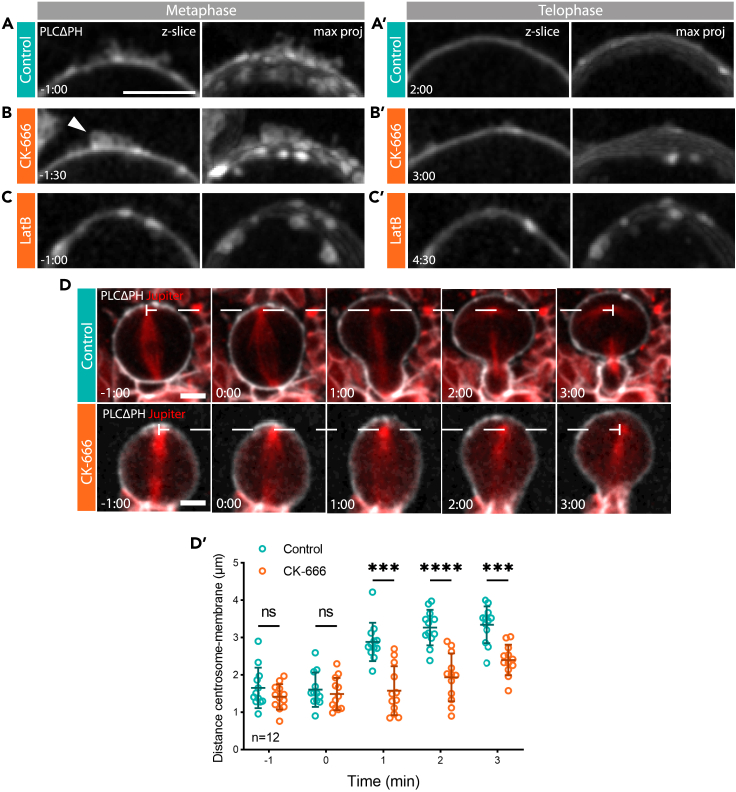
The Arp2/3 complex is required for the organization of apical actin-rich protrusions and for the precise dynamics of apical expansion (A–C′). Images depict a single z-slice together with a maximum intensity projection for the apical-most domain of neuroblasts expressing a PLCΔPH::GFP marker. Images show control (A-A′), CK-666-treated (B-B′) and Latrunculin B-treated (C-C′) cells at metaphase and telophase. (D) Images show representative control (CK-689) and CK-666 treated neuroblasts expressing a membrane marker PLCΔPH::GFP together with mCherry:Jupiter. Dotted line marks the position of the centrosome, which remains in place during mitosis. D’. Graph shows mean and standard deviation of the distance between the centrosome and apical plasma membrane in control and CK-666 treated cells as they progress through mitosis. n = 12. ns, not significant, ∗∗∗p ≤ 0.001, ∗∗∗∗p ≤ 0.0001 (t-test). Central and error bars = mean and standard deviation. Scale bars = 5 μm.

Because the Arp2/3 complex nucleates branched actin filaments downstream of SCAR in flies and other animals,^31^ to test whether these apical SCAR-rich protrusions depend on the activity of the Arp2/3 complex for their formation, we imaged neuroblasts expressing PH::GFP following treatment with the Arp2/3 inhibitor CK-666 at high spatial resolution. Although in metaphase, CK-666 treated cells possessed PH-rich membrane domains like those seen in the control, the small molecule inhibitor had a profound impact on the organization of protrusions (Figures 3A and 3B, Video 1). Instead of forming protrusions, the apical membrane was observed bulging into small rounded structures (Figures 3A and 3B, arrowhead). These data suggest that although the Arp2/3 complex activity is not required for the accumulation of apical membrane, it is required for its proper organization. Nevertheless, during the metaphase-anaphase transition, the membrane domains present in CK666-treated cells smoothed out so that they were no longer visible by cytokinesis (Figure 3B’). This occurred with similar kinetics to the loss of membrane protrusions in the control (Figure 3A’). These data suggest that the PIP_2_-rich domains flows visible in cells undergoing the metaphase-anaphase transition are not profoundly affected by the presence of an Arp2/3 inhibitor (Figure S3A).

Video S1. The Arp2/3 complex is required for the organization of apical actin-rich protrusions and for the precise dynamics of apical expansion, related to Figure 3 Video depicts a maximum intensity projection of neuroblasts expressing a PLCΔPH::GFP marker. A control cell (CK-689) is on the and a CK-666-treated cell on the right.

As additional evidence in support of a role for actin filaments in the formation of the polarized membrane protrusions in this system, we also treated cells in prophase with Latrunculin B (LatB) – a small molecule that induces the rapid loss of actin filaments by binding to actin monomers and preventing their polymerisation.^32^ Again, the addition of this actin inhibitor led to the formation of disorganized patches of apical membrane in metaphase cells (Figures 3A–3C), which remained visible through later stages of mitosis (Figure 3C’).

During the transition from metaphase to anaphase, the apical actomyosin cortex of neuroblasts has been shown to be rapidly remodeled to induce relaxation of the apical cell pole and to trigger the flow of Myosin that leads to asymmetric furrow formation. Thus, to test if Arp2/3 inhibition, as well as perturbing the formation of apical actin protrusions, has an effect on actomyosin remodeling during metaphase-anaphase transition, we measured the distance between the apical centrosome and the apical plasma membrane that undergoes cortical expansion (Figure 3D–3D′). Although the apical cortex was observed expanding quite suddenly in control cells, rapidly reaching a distance of ∼4μm from the centrosome, this movement was much less pronounced in CK-666 treated cells (Figure 3D’). This conclusion was confirmed by measuring the movement of the membrane during cortical expansion by extracting coordinates from kymographs (Figures S3B–S3B″). When the data for individual cells were plotted and averaged (Figure S3B”), it was clear that the apical membrane domain in control cells expands by undergoing a sudden movement that quickly comes to a stop. By contrast, in cells treated with the Arp2/3 inhibitor, CK-666, the rate of apical expansion was slower and occurred with near linear kinetics. As a result, a sigmoidal curve fit the control data best, whereas the CK-666 treatment curve was best modelled as a line. Thus, although CK-666 treatment does not block apical expansion, it changes the dynamics of cell shape changes following the onset of anaphase. Finally, the rapid change in the intensity of PH::GFP that occurs as the apical surface expands and smooths in control cells entering anaphase also appeared damped in cells in which Arp2/3 or SCAR complex activity were inhibited (Figures S3C–S3C′). These data suggest that SCAR likely works together with the Arp2/3 complex in regulating organization of the apical cortex in mitotic neuroblasts.

**Figure 4:**
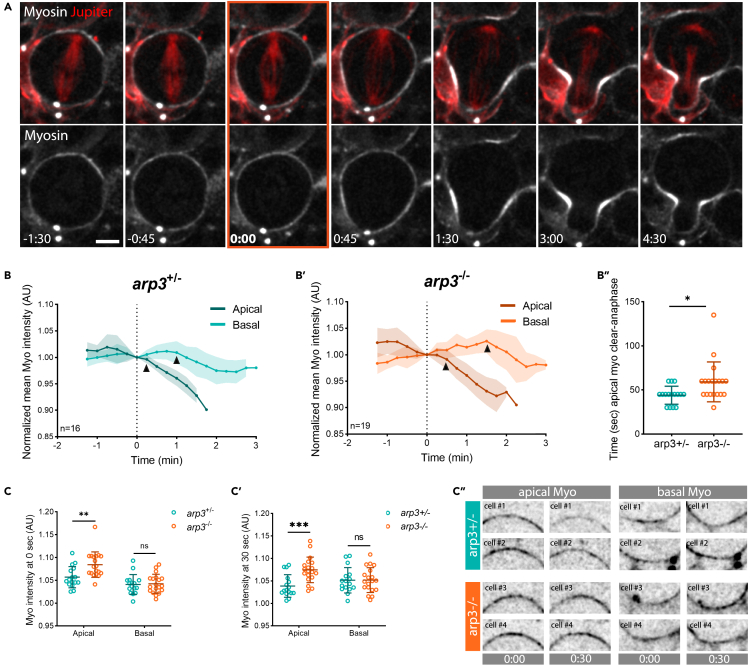
Arp2/3 modulates apical Myosin dynamics in mitotic neuroblasts (A) Super-resolution time-lapse image of dividing neuroblast expressing non-muscle Myosin II marker (Sqh:GFP) and microtubule marker (UAS-cherry:Jupiter). (B-B′). Graphs showing Myosin intensity changes during metaphase-anaphase transition in heterozygous *arp3*^*+/−*^ (arp3^EP3640^/TM6B) (B) and mutant *arp3*^*−/−*^ (arp3^EP3640^/Deficiency) cells (B′) expressing Sqh:GFP and UAS-cherry:Jupiter. Arrowheads mark the start of Myosin clearance. Myosin intensity was normalized by subtracting the background and centered at the onset of anaphase (t = 0). B”. Plot shows the timing of apical Myosin clearance relative to anaphase onset in control (arp3^EP3640^/TM6B) and (arp3^EP3640^/Deficiency) cells (t-test). (C-C′) Myosin intensity at time t = 0 (C) and t = 30 s (C′) at the apical and basal sides of *arp3*^*+/−*^ and *arp3*^*−/−*^ cells (Tukey’s multiple comparison test). Myosin intensity was normalized by subtracting background. C”. Examples of apical and basal cortex with Myosin signal at 0 and 30 s after anaphase onset for *arp3+/−* and *arp3−/−* cells. For the heterozygous control, n = 16; for the homozygous mutant, n = 19. Asterisks denote statistical significance. ns, not significant, p > 0.05, ∗p ≤ 0.05, ∗∗p ≤ 0.01, ∗∗∗p ≤ 0.001. Scale bar = 5 μm. Central and error bars = mean and standard deviation.

The polarized cortical expansion observed in neuroblasts is thought to reflect a difference in the relative timing of Myosin clearance at the apical and basal surface of cells entering anaphase.^22^^,^^23^ Because SCAR is concentrated at the apical cell cortex, which is the first to lose Myosin at the onset of anaphase to trigger an apico-basal directed cortical flow (Figure S4A), we thought it important to test whether or not SCAR and the Arp2/3 complex have a role in this process. To do so, we first imaged non-muscle Myosin II, using the Sqh:GFP reporter, at higher temporal (15 s/frame) and spatial resolution (Figure 4A). We then quantified Myosin levels at both the apical and basal sides of *arp3* mutant neuroblasts, using heterozygous animal as controls. In heterozygous animals, cortical flow resembles that seen in the wild type (Figures 4B, and S4B), with the clearance of apical Myosin beginning around 15 s after anaphase onset, followed by basal clearance 45 s later (Figure 4B, arrowheads). By contrast, there was a significant delay in the timing of Myosin clearance in homozygous *arp3* mutant animals following the onset of anaphase. This occurred at 30 s for the apical cortex, and at 90 s at the basal side (Figure 4B’, arrowheads, and 4B″). Furthermore, when comparing Myosin intensities at the onset of anaphase and 30 s later, it became clear that *arp3* mutant cells accumulate higher levels of apical cortical Myosin than their control counterparts (Figures 4C–4C″). Similarly, SCAR RNAi cells entering anaphase failed to downregulate cortical Myosin as rapidly as controls (Figure S4C). Taken together, these data suggest that the assembly of a SCAR- and Arp2/3-dependent network of actin filaments negatively regulates cortical Myosin recruitment, to promote rapid apical Myosin clearance at the onset of anaphase.

**Figure 5:**
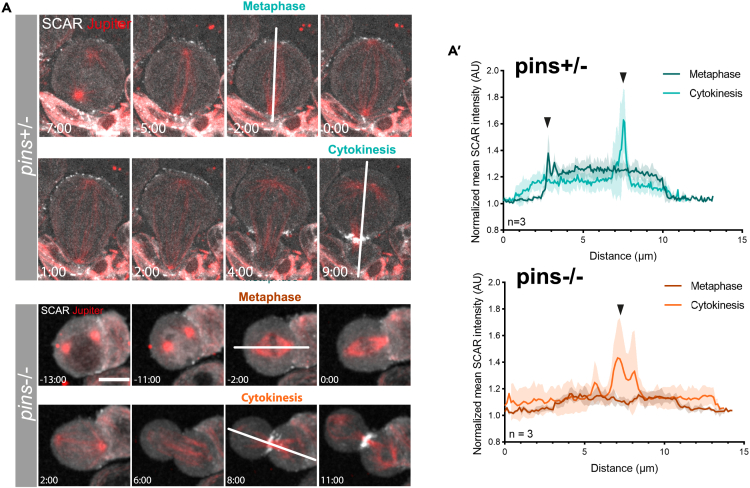
SCAR apical localization depends on the polarity protein Pins (A) Live cell imaging of neuroblasts in heterozygous (top, *pins+/−)* or homozygous (bottom, *pins*^*P62*^*/pins*^*P89*^) mutant flies expressing the SCAR reporter UAS-SCAR::GFP and the microtubule reporter UAS-cherry:Jupiter under the control of Wor-Gal4. (A′) Graphs show SCAR intensity across the cell in *pins* heterozygous and *pins* homozygous mutant cells at metaphase and cytokinesis. White lines in images in A indicate positions at which intensity measurements were made. Arrowheads show peaks of intensity. n = 3. Central and error bars = mean and standard deviation. Scale bar = 5 μm.

To determine if SCAR is recruited to the apical cortex through the set of well-established polarity cues previous work has shown operate in fly neuroblasts,^33^ we looked at SCAR localization in *pins* mutants. Pins was chosen for this analysis as it has been shown to link the polarity complex (Par-3/Par-6/aPKC) to astral microtubules. Furthermore, removing it causes neuroblasts to divide symmetrically.^21^ Although in heterozygous *pins+/−* mutant cells SCAR accumulated at the apical cortex and, later, at the cytokinetic furrow (Figures 5A–5A′), SCAR was not visible at the apical cortex of in metaphase cells homozygous for the *pins* mutant (*pins*^−/−^), which tended to be smaller and to divide symmetrically. Nevertheless, SCAR was still found accumulating at the furrow as these cells completed cytokinesis (Figures 5A–5A′). These data suggest that SCAR is recruited to the apical cortex by polarity cues established early on in the cell cycle, but is independently recruited to the cytokinetic furrow at division by other cues.

**Figure 6:**
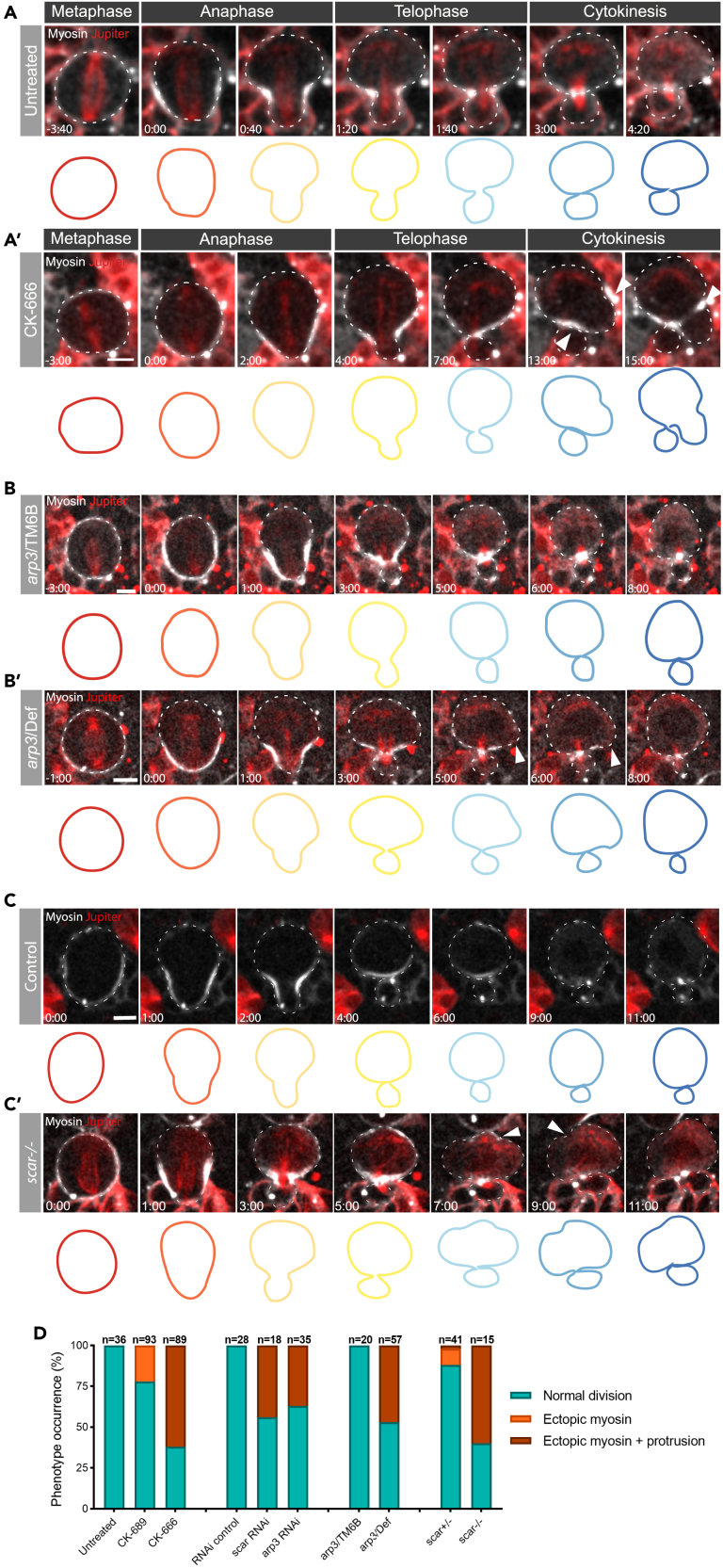
Reductions in the levels of SCAR or Arp2/3 activity lead to cortical instability as neuroblast cells divide (A–C’) Representative time-lapse images of dividing neuroblasts expressing the non-muscle Myosin II reporter Sqh:GFP together with a microtubule marker UAS-cherry:Jupiter. Examples show: Untreated (A) and CK-666 treated (A′) neuroblasts. Cells in heterozygous (*arp3*/TM6B) animals (B), and in homozygous mutant *arp3* (*arp3*/Deficiency) animals (B'). Heterozygous control (C, *scar +/-*) and s*car* mutant clones (C′, *scar−/−*) in the same tissues. Arrowheads point to defects in local cortical and membrane organization resulting from Arp2/3 or SCAR inhibition. Cell contours on the bottom show how cell shape changes from anaphase to cytokinesis for each condition. Contours are color-coded by time. (D) Graph shows the percentages of cells that appear to divide normally, versus those that divide with aberrant local cortical Myosin accumulation or with cortical shape defects, under each set of conditions. Scale bar = 5 μm.

Finally, we used live imaging to determine how the statistically significant but relatively mild changes in cortical remodeling seen in cells compromised for SCAR or Arp2/3 complex activity were likely to influence later stages in the division process. To do so, we imaged neuroblasts expressing Myosin marker Sqh:GFP and the microtubule marker UAS-cherry:Jupiter (Figure 6A), and used the Arp2/3 inhibitor CK-666 together with genetic tools (mutation and RNAi-mediated silencing of *arp3*) (Figures 6A’–6B′, 6D, and S5A) to inhibit Arp2/3 complex function. Although similar cortical defects were observed in all cases (as is evident from cell outlines in Figures 6A–6C′), the strongest phenotypes were observed following the chemical inhibition of Arp2/3. In CK-666 treated cells, but not the corresponding control, Myosin was observed ectopically accumulating at the cortex after the completion of cytokinesis, leading to an aberrant late constriction of the plasma membrane, which generated a large rounded protrusion (Figure 6A’, arrowheads and 6D). The necks of these protrusions were never seen closing, arguing against this being an ectopic cytokinetic furrow. A milder version of this phenotype was observed in cells homozygous for *arp3* mutations, and in *arp3* RNAi cells, where the accumulation of ectopic Myosin was accompanied by a range of cortical defects (Figures 6B–6B′, 6D, and S5A). In addition, similar defects were seen in somatic *scar* mutant clones in the larval brain and in cells in which *scar* was silenced using RNAi (Figures 6C–6C′, 6D and S5B). The ectopic Myosin localization and cortical defects observed after the completion of cytokinesis in cells depleted for SCAR were qualitatively similar to those observed following perturbation of the Arp2/3 complex (Figure 6C’ arrowheads and Figure S5B arrowheads) – as is evident from the montages of cell contours over time (Figures 6A–6C′). These experiments confirm a role for the SCAR and Arp2/3 complexes in regulating proper asymmetric neuroblast division, and show that their dysregulation leads to a range of cortical defects as cells progress through mitosis and divide.

## Discussion

In this study we present a role for SCAR and the Arp2/3 complex in regulating membrane organization and cell shape changes in metaphase-anaphase transition in neural stem cells in the fly. Thanks to high-resolution spinning-disk microscopy we were able to image the asymmetric accumulation of filopodia-like membrane protrusions rich in SCAR, actin and PIP_2_ lipids at the apical cortex of these cells in metaphase (Figure 1). Although Arp2/3 and SCAR complexes are usually associated with the formation of lamellipodia, this is not unexpected, because in flies these complexes have been shown to generate filopodial-like cell extensions from an underlying branched actin network in cells in culture and *in vivo.*^30^^,^^34^

Although the Arp2/3 complex is responsible for protrusion formation, we still observed an accumulation of excess apical membrane in patches following perturbations in Arp2/3 complex activity (Figure 3). This suggests that although the Arp2/3 complex nucleates an actin network which functions as a scaffold for membrane protrusion formation, it is not necessary for apical membrane accumulation (although the Latrunculin experiment suggests that actin filaments may play a role in this process).

The apical, actin-rich membrane protrusions formed as cells enter mitosis quickly disappeared as cells underwent cortical expansion as they entered anaphase (Figure 1). Although this might suggest a role for protrusions in providing a pool of excess membrane to facilitate cortical expansion (as was recently suggested by others^35^), inhibiting the Arp2/3 complex and protrusion formation did not block the accumulation of an apical pool of membrane rich in PIP_2_ as measured by PH::GFP, and did not prevent cells from completing division (Figures 5, and S3). Thus, our study does not point to protrusions acting as a functionally important membrane reservoir.

On the other hand, the lack of Arp2/3 activity was found to alter the dynamics of cortical expansion, cortical stability, and led to aberrant changes in the shape of cells undergoing cytokinesis (Figures 3 and 5). These data point to a role for the Arp2/3 complex in polarizing the cortex to regulate relaxation of the apical pole and the dynamics of shape changes that follow. In this process, the SCAR complex may provide a cue that biases the accumulation of Arp2/3 leading to the formation of a branched apical actin network.

The inhibition of Arp2/3 complex activity also led to an increase of apical Myosin at the onset of anaphase (without affecting the basal Myosin pool), and to a delay in the clearance of apical Myosin following the onset of anaphase (Figure 4). In *Drosophila* salivary glands, where the actomyosin cortex functions to drive the collapse of large spherical secretory vesicles, the Arp2/3 complex has been proposed to form stripes of branched actin that help to break the symmetry of the formin-nucleated actomyosin cortex around the vesicle to facilitate orderly vesicle contraction.^36^ We propose that SCAR and Arp2/3 complexes act in a similar way in mitotic neuroblasts to pattern the actomyosin cortex to facilitate asymmetric cell division. In this case, an apical Arp2/3 dependent actin network may limit the apical accumulation of Myosin^37^^,^^38^ to facilitate rapid apical cortical expansion at the onset of anaphase. Although it is not clear precisely how these early changes in cortical remodeling dynamics affect cytokinesis, it is possible that defects early on in the process lead to stronger phenotypes at later stages of division. Nevertheless, it remains possible that the loss of branched actin from the apical cortex has other effects on the system that alter cortical instability at later stages in other ways - for example by altering SCAR activity at the cytokinetic furrow.

Our data suggest that SCAR is likely to be the main Arp2/3 activator in the generation of apical membrane protrusions in this system, because reduction in SCAR (using mutants and RNAi) led to the same class of phenotypes as those observed following reduction in Arp2/3 activity (drugs, mutants and RNAi) (Figure 5). Although this is the case, the membrane constriction phenotype observed at cytokinesis was stronger and more consistent in CK-666 treated cells than it was following perturbations of SCAR function. Because flies possess additional NPFs, it is also possible that WASH and WASp play minor roles as Arp2/3 activators in this system. This is often the case with NPFs. Thus, although there tends to be a clear separation between the functions of Arp2/3 nucleation promoting factors, with SCAR being responsible for lamellipodia formation, WASp being involved in filopodia, and WASH being involved in trafficking, this is not always the case.^31^^,^^39^ If they have partially redundant roles, this may explain why the Arp2/3 phenotypes tend to be stronger than those seen following reductions in SCAR levels.

At the same time, the SCAR depletion phenotype is clear and fits with SCAR's previously described role in the formation of thin actin-based protrusions.^9^^,^^30^^,^^40^ Moreover, SCAR appears to be in the right place at the right time to generate apically polarized Arp2/3 dependent protrusions. We also show that cortical SCAR is recruited through polarity cues like Pins proteins. A more extended analysis of the other polarity proteins, like Par-3, Par-6 and aPKC, would likely shed light on how the complex is recruited and its activity regulated. In both *Drosophila* neuroblasts and *C. elegans,* a link between these proteins and F-actin, Myosin and membrane domains has been clearly established.^24^^,^^26^^,^^41^ Our data also aligns with recent work done in *Drosophila* SOP cells where Arp2/3 and SCAR complexes were shown to regulate daughter cell size in an asymmetric division^13^ and with a study carried out in parallel on cortical remodelling and membrane flows in neuroblasts.^35^ In general, therefore, our analysis shows how the polarized localization of SCAR locally activates Arp2/3 to break the symmetry of the cortical actomyosin network in metaphase. At the onset of anaphase, the presence of an apical branched actin network may then help to tune the actomyosin cortex to enable the precise control of changes in cell shape and membrane organization required for asymmetric cell division.

### Limitations of the study

Using available methods, we were unable to quantify local levels of branched and unbranched actin in normal and perturbed conditions in dividing neuroblasts. Although determination of the precise structure of the apical actin cortex would require CryoEM, something that is currently impossible in this system, because SCAR and the Arp2/3 complex work together to nucleate branched actin networks across systems, our data strongly suggest a role for branched actin in asymmetric cortical expansion. In addition, because we were unable to specifically test the functions of accumulations of SCAR and Arp2/3 complexes at the cytokinetic furrow, it is possible that this late stage recruitment plays the major role in the regulation of cortical stability after division. A further limitation of this study is the absence of phenotypic data for loss of function perturbations of upstream activators such as Rac, and the alternative Arp2/3 activators, WASp and WASH, since we were unable to obtain unequivocal data when testing their contribution to cortical dynamics during cell division in this system. Finally, although we were unable to directly test the impact of SCAR and Arp2/3 nucleated actin on Myosin II's ability to remodel an actin network in this system, our data are consistent with observations made by others using simpler systems.^37^^,^^38^

## STAR★Methods

### Key resources table

**Table:** 

REAGENT or RESOURCE	SOURCE	IDENTIFIER
**Experimental models: Organisms/strains**		

Arp3^EP3640^	(Rørth 1996)	RRID:BDSC_17149
Df(3L)Exel6112	(Parks et al. 2004)	RRID:BDSC_7591
SCAR^Δ37^ FRT40A	(Zallen et al. 2002)	RRID:BDSC_8754
SCAR RNAi	(Perkins et al. 2015)	RRID:BDSC_36121
Sqh:GFP	(Royou, Sullivan, and Karess 2002)	FBti0073027
UAS-mCherry:Jupiter	(Cabernard and Doe 2009)	FBtp0040573
UAS-PLCΔPH::GFP	N/A	RRID:BDSC_39693
UAS-PH:mCherry	N/A	RRID:BDSC_51658
UAS-LifeAct:GFP	N/A	RRID:BDSC_58718
UAS-SCAR::GFP	From M. González-Gaitán	N/A
worniu-Gal4	(Albertson and Doe 2003)	FBti0161165

**Chemicals, peptides, and recombinant proteins**		

CK-689	Sigma	SML0006
CK-666	Sigma	182517
LatrunculinB	Sigma	L5288

**Software and algorithms**		

ImageJ 1.52p	(Schindelin et al. 2012)	RRID:SCR_003070
GraphPad	N/A	RRID:SCR_000306
Adobe Illustrator CS6	N/A	RRID:SCR_010279

### Resource availability

#### Lead contact

Further information and requests for resources and reagents should be directed to and will be fulfilled by the lead contact, Buzz Baum (bbaum@mrc-lmb.cam.ac.uk)

#### Materials availability

This study did not generate new unique reagents.

### Experimental model and study participant details

#### Fly strains and genetics

Flies were raised in vials containing standard cornmeal-agar medium supplemented with baker’s yeast. Crosses were incubated at 25°C and larvae were grown for 4 days before being dissected for live imaging. Mutant chromosomes were balanced over Cyo:ActGFP or TM6B, Tb. The following mutant alleles and RNAi lines were used: Arp3^EP3640^ (BL17149, Bloogminton),^42^ Df(3L)Exel6112 (removes Arp3, BL7591, Bloogminton), SCAR^Δ37^ FRT40A (BL8754, Bloomington),^40^ SCAR RNAi (BL36121, Bloomington). The following transgenes and fluorescent markers were used: Sqh:GFP,^43^ and UAS-cherry:Jupiter^44^ from C. Roubinet. UAS-PLCΔPH::GFP (BL39693, Bloomington), UAS-PH:mCherry (BL51658, Bloomington), UAS-LifeAct:GFP (BL58718, Bloomington), UAS-SCAR::GFP (from M. González-Gaitán). Transgenes were expressed using the neuroblast-specific driver worniu-Gal4.^45^

### Method details

#### Live imaging sample preparation

Larvae were dissected to extract the brains in imaging medium (Schneider’s insect medium mixed with 10% FBS (Sigma), 2% PenStrepNeo (Sigma), 0.02 mg/mL insulin (Sigma), 20mM L-glutamine (Sigma), 0.04 mg/mL L-glutathione reduced (Sigma) and 5 μg/mL 20-hydroxyecdysone (Sigma)). Brains were then transferred with the medium onto 15μm-slide angiogenesis (Ibidi), brain lobes facing down, and imaged. When brain dissociation was performed, larvae were dissected in Chan & Gehring solution 2% FBS (CG-FBS) to extract the brain (Chan & Gehring, 1971). GC-FBS composes as follow: NaCL 3.2 g/L, KCl 3 g/L, CaCl_2_-2H_2_O 0.69 g/L, MgSO_4_-7H_2_O 3.7 g/L, Tricine buffer Ph7 1.79 g/L, glucose 3.6 g/L, sucrose 17.1 g/L, BSA 1 g/L and FBS 2%. Papain (Sigma, #P4762-50MG, 10 mg/mL) and collagenase (Sigma, #C2674-1G, 10 mg/mL) were added to the brains in CG-FBS solutions and they were incubated at 29°C for 45 min, to activate the enzymes. After incubation, brains were washed with imaging medium and finally dissociated through vigorous pipetting. The brains were then transferred with the medium onto 15μm-slide 8 well (Ibidi) and imaged.

#### Imaging

Super-resolution imaging was performed on a CSU-W1 SoRa spinning disk confocal microscope (Nikon Ti Eclipse 2; Yokogawa CSU-W1 SoRa spinning disk scan head) with 60×/1.40 N.A oil objective and equipped with a photometrics prime 95B scientific CMOS camera. Whole brain live imaging was performed on an UltraView Vox spinning disk confocal microscope (Perkin Elmer Nikon TiE; Yokogawa CSU-X1 spinning disc scan head) with 60×/1.40 N.A oil objective and equipped with a Hamamatsu C9100-13 EMCCD camera. Whole brain imaging has been acquired with a z stack spacing of 1 μm and have a spatial resolution of 5.4 pixel per micron, whereas single cell imaging with a spacing of 0.7 μm with a spatial resolution of 7.6 pixel per micron. Time resolution was 60 s per frame, unless specified otherwise. Both microscopes are equipped with a temperature-controlled environment chamber set at 26°C for the experiments.

#### Treatments

For chemical treatments to inhibit the Arp2/3 complex, the inhibitor CK-666 (Sigma #SML0006, final concentration 400 μM) or the inactive equivalent compound, CK-689 (Sigma #182517, final concentration 400 μM), were added before live imaging. To induce actin depolymerization, Latrunculin B (Sigma #L5288-1MG) at a final concentration of 10 μM was added to the media during live imaging.

### Quantification and statistical analysis

#### Image processing

All image analysis was carried out on unprocessed raw images. For clarity, images displayed in this work were processed using ImageJ software.^46^ Background was subtracted (rolling ball radius 50 pixel) and a Gaussian Blur applied (radius 1). As stated in Figure legends, images represent a single confocal z stack section or a maximum z-projection. In all figures, the time point 0 is anaphase onset, defined in this work as the first frame in which the spindle is observed elongating. Figures were assembled using Adobe Illustrator CS6.

#### Image analysis

Experiments in which cortical PH or Myosin intensity were measured, a line was drawn over the area of interest and the mean pixel value calculated. The data were normalized by subtracting the background and were centered at the earliest time point. To calculate the movement of the membrane during cortical expansion, a maximum projection of 3 z-slices from the center of cells dividing along the axis parallel to the field of view was generated. A line was drawn from the center of the spindle to the apical membrane, starting two time-frames before anaphase onset. A kymograph was generated from this line, the movement of the membrane was traced and the set of coordinates were used to generate the curves in the graphs. Coordinates were centered to start at (x = 0, y = 0). To plot the mean, the curves were interpolated in Excel using linear interpolation.

#### Statistical analysis

For quantifications, data was collected from at least 2 independent experiments, and, for each independent experiment, at least 2 brain lobes were imaged. For the analysis, “n” refers to the number of cells analyzed and is represented on the graph or mentioned in figure legends. Statistical significance was determined with Student’s *t* test where two groups were compared, or two-way ANOVA followed by post hoc Tukey’s multiple comparison where more than two groups were compared, using GraphPad Prism 9 software. In supplemental Figure S3B”, fits to both control and treatment curves were carried out using linear regression and a sigmoid function (model: Y=Bottom + (Top-Bottom)/(1 + 10ˆ((LogEC50-X)∗HillSlope))). In all figures the Prism convention is used: not significant (p > 0.05), ∗(p ≤ 0.05), ∗∗(p ≤ 0.01), ∗∗∗(p ≤ 0.001) and ∗∗∗∗(p ≤ 0.0001). In all graphs showing mean, the error bars correspond to standard deviation (SD).

## Data Availability

Data reported in this paper will be shared by the lead contact upon request. This paper does not report original code. Any additional information required to reanalyze the data reported in this paper is available from the lead contact upon request.
