# Supplementary material for: Polarized SCAR and the Arp2/3 complex regulate apical cortical remodeling in asymmetrically dividing neuroblasts
Source: iScience. 2023 Jun 15;26(7):107129. doi: 10.1016/j.isci.2023.107129 (PMC10331462; doi:10.1016/j.isci.2023.107129)
Supplement: Document S1 Figures S1–S5 [file mmc1.pdf]

iScience, Volume *26*

Supplemental information

Polarized SCAR and the Arp2/3 complex regulate apical cortical remodeling in asymmetrically dividing neuroblasts

Giulia Cazzagon, Chantal Roubinet, and Buzz Baum

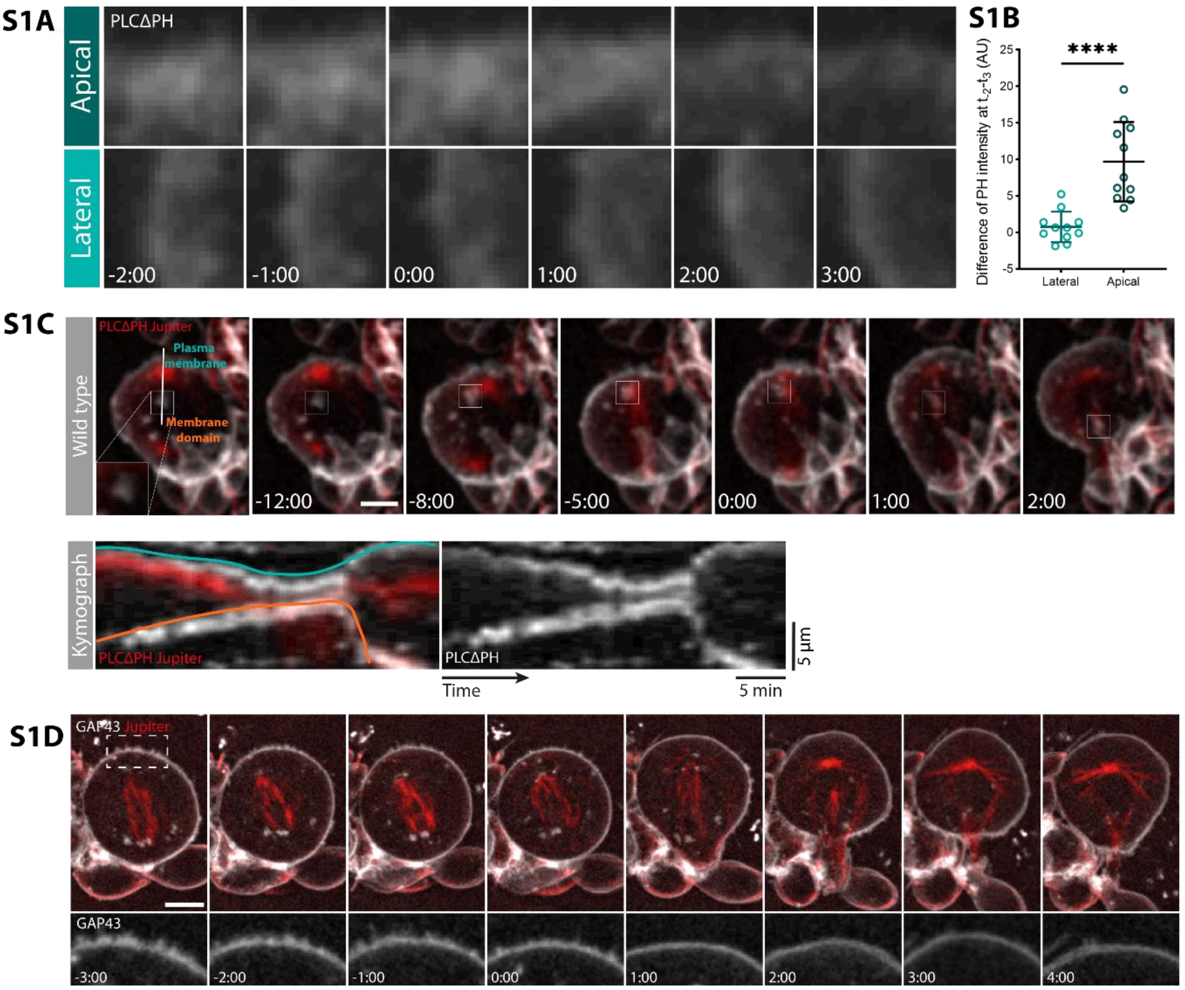

Figure S1. The apical plasma membrane is remodelled as neuroblasts progress through mitosis, related to Figure 1. A. Images show a zoom in of the apical and lateral membrane of a wild-type neuroblast expressing UAS-PLCΔPH::GFP. **B.** Plot showing difference of PH intensity between t = -2min and t = 3min in both lateral and apical membrane domains. Asterisk (****) denote statistical significance. P ≤ 0.0001 (paired t-test). Central and error bars: mean and SD. **C.** Maximum intensity z-projection of dividing neuroblast expressing membrane marker, UAS-PLCΔPH::GFP and a microtubule marker UAS-mCherry::Jupiter, both expressed via Wor-GAL4/UAS. The white line indicates the position used to generate the kymograph. Insert shows an example of a membrane domain. Kymograph shows the movement of the plasma membrane (blue) and of a more basal PH::GFP-rich membrane domain (orange). **D.** High resolution imaging of neuroblast expressing membrane marker, mCherry::GAP43, driven by sqh promoter. Insert shows membrane protrusions at the apical side of the cell in metaphase (-3:00 to -1:00 min). Apical protrusions begin to disappear as cells enter anaphase. Scale bars = 5 µm.

S2

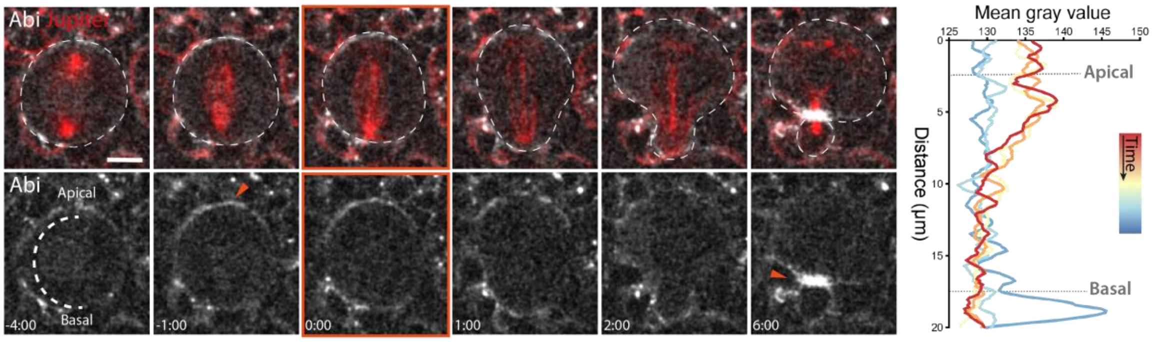

Figure S2. A second component of the SCAR complex, Abi, localizes at the apical side of the neuroblast at metaphase and the furrow at cytokinesis, related to Figure 2. Representative super-resolution images of neuroblast expressing *ubi*-mCherry::Abi. Arrowheads point to Abi localization at the apical cortex in metaphase and at the furrow at cytokinesis. Mean Abi signal intensity at each timepoint was acquired by drawing a line around the cortex from the apical to the basal side, as depicted at time -4:00 relative to anaphase onset. These values were then plotted in graphs on the right. Scale bar = 5 µm.

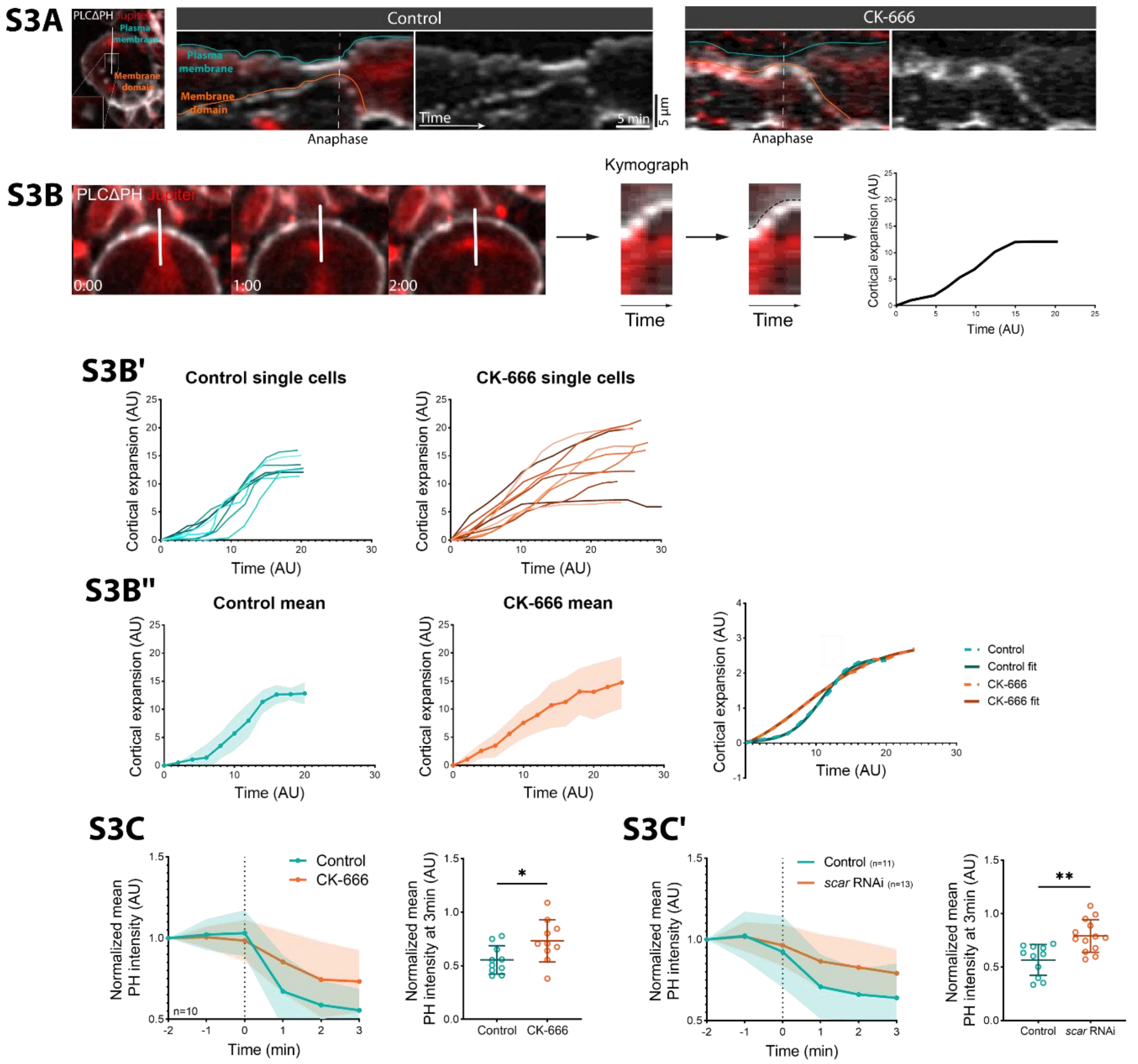

Figure S3. Arp2/3 and SCAR are required for the normal remodelling of the apical membrane in neuroblasts at the onset of anaphase, related to Figure 3. A. Representative kymographs of control (CK-689 treatment) and CK-666 treated cells expressing PLCΔPH::GFP and mCherry::Jupiter as they progress from prophase to cytokinesis. Lines indicate the movement of the apical plasma membrane (blue lines) and the movement of PLCΔPH::GFP punctae (orange lines). The cell shown on the left is an example of a maximum intensity z-projection of a dividing neuroblast in which the white line indicates the position used to generate the kymograph. **B.** Schematics showing how the kymograph and plots in B'-B" were obtained. In brief, a line drawn on the movie was used to generate a kymograph from which coordinates were manually tracked, exported and plotted to follow the movement of the membrane (graph on the right). **B'-B".** Graphs show single cells tracks (**B'**) and mean path (**B"**) of membrane expansion during anaphase for control (CK-689) and CK-666 treated cells. Coordinates were centred to start at (x=0, y=0), and a linear interpolation of the x set of coordinates was performed. Graphs on the right show curves for mean cortical expansion for control and CK666 treated cells with their fitted curves: the control curve is fitted best by a linear regression, and the treatment curve is fitted best by a sigmoid. n=10. **C.** Plots show changes in PLCΔPH::GFP intensity during metaphase-anaphase transition in control (CK-689) and CK-666 treated cells, and statistically significant difference between PH intensity at the last time point (3 min). n=10. t-test: *P ≤ 0.0001. **C'.** Plot showing changes in PLCΔPH::GFP intensity during metaphase-anaphase transition in Control and Scar RNAi cells, and statistically significant difference between PH intensity at the last time point (3 min). n control=11, n RNAi=13. T-test: **P ≤ 0.01. Central and error bars = mean and standard deviation.

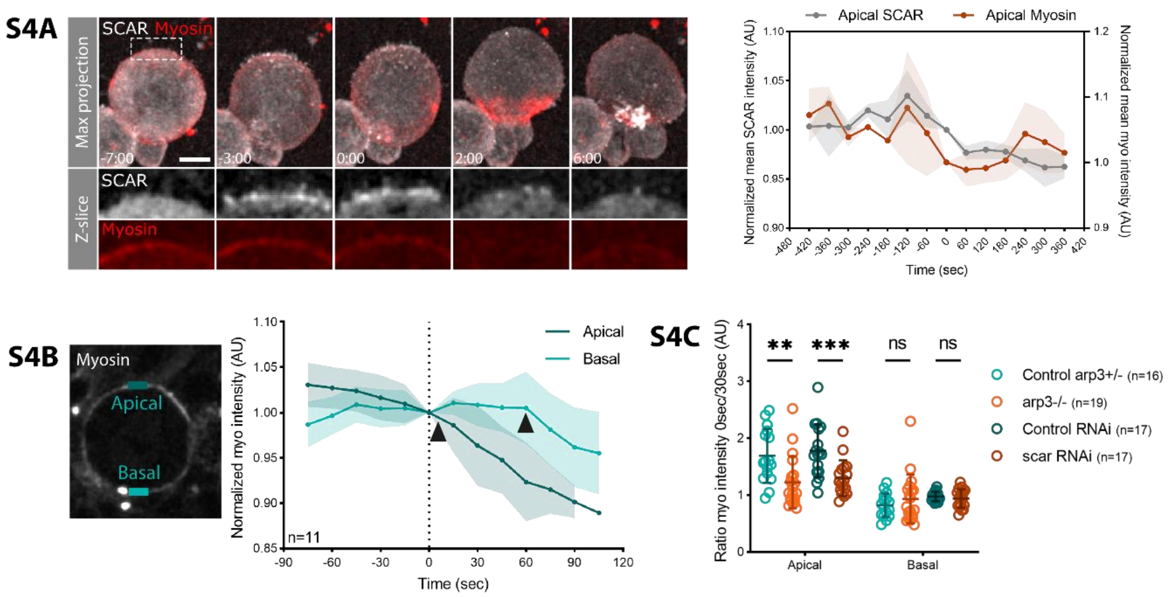

Figure S4. Apical Myosin dynamics at the metaphase-anaphase transition, related to Figure 4. **A.** Representative images of dissociated neuroblast cells expressing both UAS-SCAR::GFP and the non-muscle Myosin II marker, UAS-Sqh::cherry, under the control of Wor-GAL4. Inserts show apical SCAR and Myosin signals separately. Graph on the right shows SCAR and Myosin apical intensities during neuroblast division, with anaphase onset indicated by t=0. **B.** Graph shows changes in apical and basal Myosin intensity before and after anaphase onset (t=0) measured in cells expressing the Myosin marker, Sqh::GFP. Arrowheads mark the approximate times at which Myosin starts to be cleared. **C.** Graph shows the ratio of Myosin intensity at anaphase onset (0 sec) versus 30 seconds later in both apical and basal domains in CK-666 treated cells, and SCAR RNAi cells and in their respective controls (Tukey's multiple comparison test). ns, not significant, P > 0.05, **P ≤ 0.01, ***P ≤ 0.001. Scale bar = 5 µm. Central and error bars = mean and standard deviation.

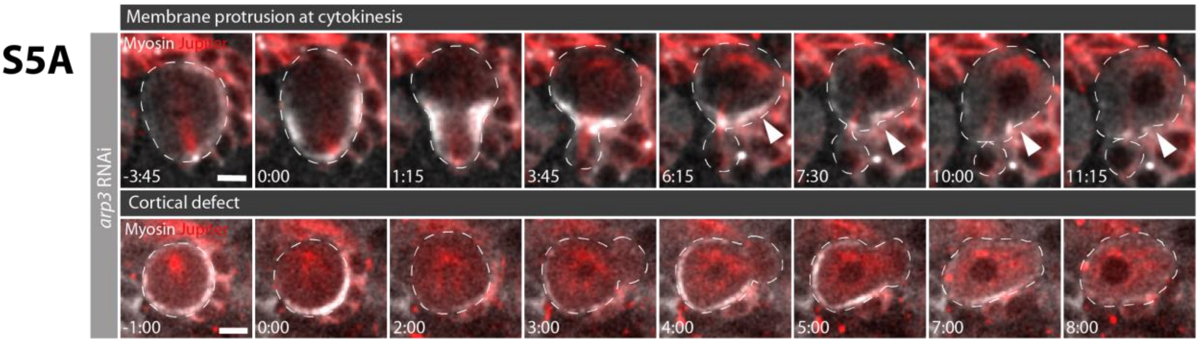

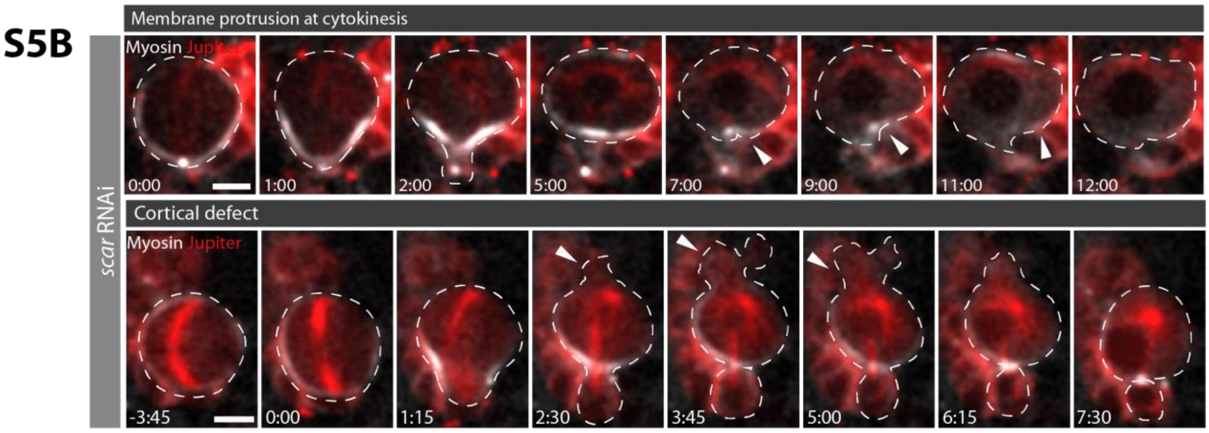

Figure S5. RNAi mediated silencing of either *arp3* or *scar* leads to cortical defects and membrane instability in neuroblasts undergoing cytokinesis, related to Figure 5. Time-lapse image of representative neuroblasts expressing the Myosin marker Sqh::GFP along with the microtubule marker cherry::Jupiter. **A.** Cells expressing dsRNA targeting the *arp3* subunit of the Arp2/3 complex. **B.** Cells expressing dsRNAs targeting *scar*. Panels on top show an example of a membrane protrusion phenotype at cytokinesis. Panels on the bottom show example of cortical defects, including blebbing.

Scale bar = 5 μm.
